# Supplementary figures and images for: The relationship between hot flashes and fatty acid binding protein 2 in postmenopausal women
Source: PLoS One. 2022 Oct 19;17(10):e0276391. doi: 10.1371/journal.pone.0276391 (PMC9581385; doi:10.1371/journal.pone.0276391)

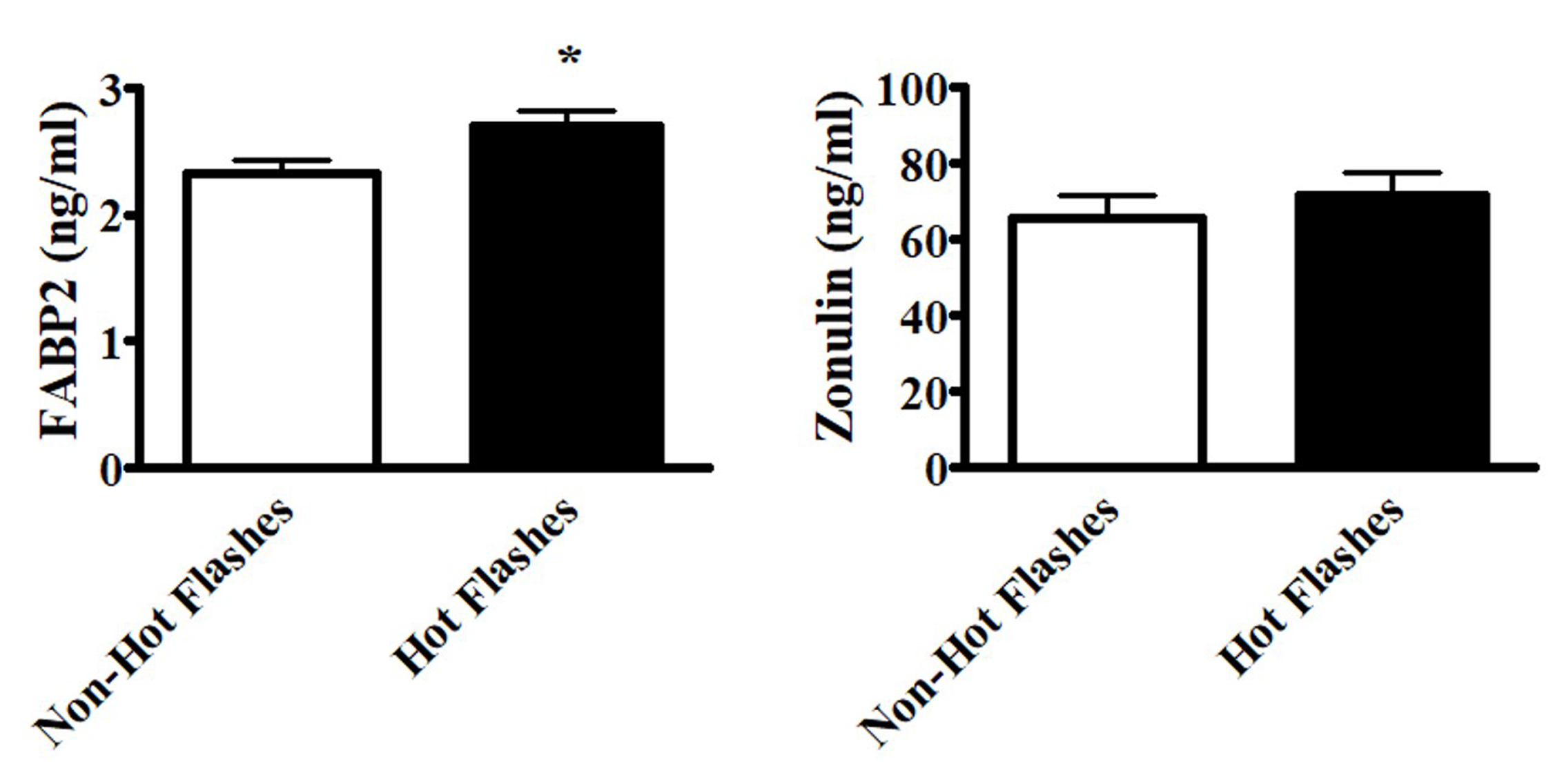

Supplement: S1 Fig — The plasma levels of gut barrier protein FABP2, but not zonulin are higher in postmenopausal women with hot flashes (n = 172) than those in women without hot flashes (n = 117) (Student’s t-test; P = 0.014). (TIF) [file pone.0276391.s001.tif]
