# Supplementary material for: The relationship between hot flashes and fatty acid binding protein 2 in postmenopausal women
Source: PLoS One. 2022 Oct 19;17(10):e0276391. doi: 10.1371/journal.pone.0276391 (PMC9581385; doi:10.1371/journal.pone.0276391)
Supplement: Attachment — Submitted filename: 00. Response to Reviewers-R1.docx [file pone.0276391.s002.docx]

PONE-D-21-34256

The Relationship between Hot Flashes and Fatty Acid Binding Protein 2 in Postmenopausal Women

**Response to reviewers’ comments:**

We would like to thank the academic editor and reviewers for the comprehensive assessment of our manuscript, giving very constructive criticism and comments. We have taken all the remarks into account, in a manner that is described in detail below together with our response to certain comments. We think that, following the reviewers’ suggestions, our manuscript has gained in clarity and hope that the changes made will be considered satisfactory.

Reviewers' comments:
Reviewer's Responses to Questions

**Comments to the Author**

1. Is the manuscript technically sound, and do the data support the conclusions?
The manuscript must describe a technically sound piece of scientific research with data that supports the conclusions. Experiments must have been conducted rigorously, with appropriate controls, replication, and sample sizes. The conclusions must be drawn appropriately based on the data presented.

Reviewer #1: Yes

Reviewer #2: Yes

 2. Has the statistical analysis been performed appropriately and rigorously?

 Reviewer #1: Yes

Reviewer #2: Yes

 3. Have the authors made all data underlying the findings in their manuscript fully available?

The [PLOS Data policy](http://www.plosone.org/static/policies.action#sharing) requires authors to make all data underlying the findings described in their manuscript fully available without restriction, with rare exception (please refer to the Data Availability Statement in the manuscript PDF file). The data should be provided as part of the manuscript or its supporting information, or deposited to a public repository. For example, in addition to summary statistics, the data points behind means, medians and variance measures should be available. If there are restrictions on publicly sharing data—e.g. participant privacy or use of data from a third party—those must be specified.

 Reviewer #1: Yes

Reviewer #2: Yes

 4. Is the manuscript presented in an intelligible fashion and written in standard English?

PLOS ONE does not copyedit accepted manuscripts, so the language in submitted articles must be clear, correct, and unambiguous. Any typographical or grammatical errors should be corrected at revision, so please note any specific errors here.

 Reviewer #1: Yes

Reviewer #2: No

**Response:**

1. We thank you very much for useful suggestion.
2. The manuscript has been revised by a native English speaker with extensive experience in medical and scientific editing.

**5. Review Comments to the Author**

Please use the space provided to explain your answers to the questions above. You may also include additional comments for the author, including concerns about dual publication, research ethics, or publication ethics. (Please upload your review as an attachment if it exceeds 20,000 characters)

Reviewer #1: 1. The results of the study evidence the association between hot flashes and gut permeability and low-grade systemic inflammation. The authors also propose the possible causal relationships between dysbiosis, gut permeability, related inflammation and hot flashes in menopausal women. This raises a concern about the study design. Do you exclude the postmenopausal patients with GI diseases, such as diarrhea, constipation, liver diseases, biliary tract diseases, inflammatory bowel diseases? Because these conditions may cause disrupted gut permeability and systemic inflammation. And they make bias in the results. Please explain and discuss it in the revised manuscript.

**Response**

1. Thank the reviewer very much for the critical comments.
2. The mechanisms of action for leaky gut are still not fully determined. Multiple contributing factors are most likely involved in the increased gut permeability, including chronic constipation, chronic bowel disorders, and hepatic biliary tract diseases (Fukui, Inflamm Intest Dis. 2016;1:135-145; Vanuytsel, Front Nutr. 2021;8:717925; Hanning, Therap Adv Gastroenterol. 2021;14:1756284821993586;	Ohkusa, Front Med. 2019;6:19) as the Reviewer’s mention.
3. We have carefully reviewed the medical history of our cases based on the reviewer’s comments. Among those subjects in the present study, there was no obvious medical history of acute and chronic bowel disorders. Their hs-CRP values were less than 10 mg/dl (Table 1), indicating no high-degraded systemic inflammation and bacterial infections. However, the constipation and diarrhea symptoms in these subjects could not be detected in detail, which may make bias in the present results.
4. According to your nice suggestions, several sentences representing the limitations have been mentioned and added into the limitation paragraph (Please see Page 16).

Reviewer #2: The authors study the relationship between hot flashes (and their severity) and gut permeability. Their study is innovative and well carried out.
I do have a few comments.
In the introduction the authors write: To gain a better understanding of the relationship between hot flashes and
gut-microbiota-derived mechanisms, a case-control study was conducted in age-matched groups
of postmenopausal women with and without hot flashes.
a - this study is cross sectional and not a case control
b - I suggest to more clearly define the objective (as defined in the discussion section) to investigate the relationship between hot flashes and gut permeability

**Response**

1. Thank the reviewer very much for very variable suggestions.
2. These points have been modified in the Introduction section (Please see Page 6).

Materials and methods: It is not clear at what time period where the women recruited (years and months). It is also not clear if all postmenopausal women who attended the hospital were offered participation? Visitors? Patients? Women attending a specific clinic?
The authors write: Linear regression analysis revealed that the severity of hot flashes had significant effects on FABP2
I recommend writing association as no causality and cause effect can be concluded
**Response**

1. Thank the reviewer very much for very useful suggestions.
2. We have added the information of the participants, who visited for health management reasons in the section of Materials and Methods (Please see Page 7).
3. In addition, the sentence “Linear regression analysis revealed that the severity of hot flashes had significant effects on FABP2” has been modified as “Linear regression analysis revealed that the severity of hot flashes had significant associations with the FABP2 plasma levels (P-trend = 0.002), but not with the zonulin levels” in the Result section (Please see Page 12).

Discussion
The authors write: "In the present study, we found that plasma levels of zonulin and FABP2 (biomarkers of gut permeability) were higher in women with hot flashes (n = 172) than in women without hot flashes (n = 117)(S1 Fig), suggesting that hot flashes were possibly related to gut permeability changes".
However, no association was found with zonulin levels.
Also - the association between FAB2 and hot flashes was found groups HFs whereas in HFM and HFm the association was not significant (p>0.05)

**Response**

1. Thank the reviewer very much for very variable comments.
2. There is a mistake in this sentence “In the present study, we found that plasma levels of zonulin and FABP2 (biomarkers of gut permeability) were higher in women with hot flashes (n = 172) than in women without hot flashes (n = 117)(S1 Fig), suggesting that hot flashes were possibly related to gut permeability changes". It has been correlated as “--- plasma levels of FABP2, but not zonulin, were higher in women with hot flashes (n = 172) than in women without hot flashes (n = 117)(S1 Fig),---.” We also modified several sentences in this paragraph (Please see this paragraph in the Discussion section, Page 13)
3. Linear regression analysis found that FABP2 levels had positive tendency to the severity of hot flashes, and multiple linear regression analysis further revealed the significant relationships between “severe” hot-flash (HFs) group and FABP2 levels. According to your nice suggestions, several sentences have been modified in the paragraph of “FABP2 Levels are independently associated with hot flashes” (Please see this paragraph in the Result section, Page 12).

Please clarify the term metacardiovascular diseases

I am not sure if this paragraph is not clear grammatically or for some other reason - but this study too (as far as I understood) did not check for an association between dysbiosis/ gut microbiota and hot flashes. Rather it checked the association with gut permeability.
**Response**

1. Thank the reviewer very much for very useful suggestions.
2. This mistake has been modified as “cardiovascular and metabolic disorders” (Please see Page 15), which includes cardiovascular diseases and metabolic diseases, such as type 2 diabetes.
3. We agree with the Reviewer’s comment. The present study did not check for an association between dysbiosis/ gut microbiota and hot flashes. Therefore, this paragraph has been modified (Please see this paragraph in the Discussion section, Pages 14 and 15)

A few typos and grammatical errors are present - please spell check and also check for grammar

**Response:**

1. We thank you very much for useful suggestion.
2. The manuscript has been revised by a native English speaker with extensive experience in medical and scientific editing.

 6. PLOS authors have the option to publish the peer review history of their article ([what does this mean?](https://journals.plos.org/plosone/s/editorial-and-peer-review-process#loc-peer-review-history)). If published, this will include your full peer review and any attached files.


If you choose “no”, your identity will remain anonymous but your review may still be made public.


Do you want your identity to be public for this peer review? For information about this choice, including consent withdrawal, please see our [Privacy Policy](https://www.plos.org/privacy-policy).

 Reviewer #1: Yes: Dr. Tsung-Hsuan Lai, M.D., Ph.D.

Reviewer #2: No

[NOTE: If reviewer comments were submitted as an attachment file, they will be attached to this email and accessible via the submission site. Please log into your account, locate the manuscript record, and check for the action link "View Attachments". If this link does not appear, there are no attachment files.]

While revising your submission, please upload your figure files to the Preflight Analysis and Conversion Engine (PACE) digital diagnostic tool, <https://pacev2.apexcovantage.com/>. PACE helps ensure that figures meet PLOS requirements. To use PACE, you must first register as a user. Registration is free. Then, login and navigate to the UPLOAD tab, where you will find detailed instructions on how to use the tool. If you encounter any issues or have any questions when using PACE, please email PLOS at [figures@plos.org](mailto:figures@plos.org). Please note that Supporting Information files do not need this step.
