# Supplementary material for: The relationship between hot flashes and fatty acid binding protein 2 in postmenopausal women
Source: PLoS One. 2022 Oct 19;17(10):e0276391. doi: 10.1371/journal.pone.0276391 (PMC9581385; doi:10.1371/journal.pone.0276391)
Supplement: Attachment — Submitted filename: 00. Response to Reviewers-R2a.docx [file pone.0276391.s003.docx]

PONE-D-21-34256R1

The Relationship between Hot Flashes and Fatty Acid Binding Protein 2 in Postmenopausal Women

**Response to reviewers’ comments:**

We would like to thank the academic editor and reviewers for the comprehensive assessment of our manuscript, giving very constructive criticism and comments. We have taken all the remarks into account, in a manner that is described in detail below together with our response to certain comments. We think that, following the reviewers’ suggestions, our manuscript has gained in clarity and hope that the changes made will be considered satisfactory.

Reviewers' comments:
Reviewer's Responses to Questions

**Comments to the Author**
1. If the authors have adequately addressed your comments raised in a previous round of review and you feel that this manuscript is now acceptable for publication, you may indicate that here to bypass the “Comments to the Author” section, enter your conflict of interest statement in the “Confidential to Editor” section, and submit your "Accept" recommendation.

Reviewer #2: All comments have been addressed

Reviewer #3: (No Response)

2. Is the manuscript technically sound, and do the data support the conclusions?

The manuscript must describe a technically sound piece of scientific research with data that supports the conclusions. Experiments must have been conducted rigorously, with appropriate controls, replication, and sample sizes. The conclusions must be drawn appropriately based on the data presented.

Reviewer #2: Yes

Reviewer #3: Yes

3. Has the statistical analysis been performed appropriately and rigorously?

Reviewer #2: Yes

Reviewer #3: Yes

4. Have the authors made all data underlying the findings in their manuscript fully available?

Reviewer #2: Yes

Reviewer #3: Yes

5. Is the manuscript presented in an intelligible fashion and written in standard English?

PLOS ONE does not copyedit accepted manuscripts, so the language in submitted articles must be clear, correct, and unambiguous. Any typographical or grammatical errors should be corrected at revision, so please note any specific errors here.

Reviewer #2: Yes

Reviewer #3: Yes

6. Review Comments to the Author

Please use the space provided to explain your answers to the questions above. You may also include additional comments for the author, including concerns about dual publication, research ethics, or publication ethics. (Please upload your review as an attachment if it exceeds 20,000 characters)

Reviewer #2: (No Response)

Reviewer #3: The authors showed that there is an association with hot flashes and gut permeability/inflammation.
The major concern with this paper is the measurement of endotoxin. Endotoxin is very difficult to measure because there is contamination of it everywhere, therefore it is almost imposible to measure endotoxin unless the samples are collected in an endotoxin free environment and are measured in an endotoxin free facility. Therefore, with the data presented in this paper it will be hard to conclude that there is increased gut permeability just by measuring FABP2. There are other ways to measure presence of microbial translocation such as LBP (LPS binding protein) or sCD14 (receptor of LPS). Therefore the conclusions made by the authors are weakened by this flaw. The conclusions will be strengthened if other markers of gut permeability/microbial translocation in addition of FABP2 are measured. Nonetheless, it is interesting that inflammation is also associated with hot flashes however the connexion of inflammation with gut permeability is weak.

**Response:**

1. Thank the reviewer very much for the critical comments and suggestions.
2. We agree with the concerns as the reviewer mentioned. Endotoxin is very difficult to measure because there is contamination of it everywhere, leading to unreliable experimental results in diagnostic and research laboratories.
3. In our Lab, we have a lot of experience in endotoxin assay and endotoxin experiments (Wu CL et al., PLoS One. 2014 Oct 6;9(10):e109558; Chang CC et al., Sci Rep. 2015;5:10096; Shih KL et al., Sci Rep. 2016;6:24031; Huang WY et al., J Neuroinflammation. 2022 Feb 2;19(1):29).
4. In our Lab, Chromogenic Limulus Amebocyte Lysate Assay (QCL-1000™; Lonza, Walkersville, MD, USA) was used for endotoxin detection. From sample collection to endotoxin detection, each procedure was performed in an endotoxin free facility, by using endotoxin-free materials and reagents, including nonpyrogenic plasticware, depyrogenated glassware, and high-purity water. These steps will prevent the most common sources of endotoxin contamination and ensure the reliability of the obtained results. Therefore, several sentences representing the concern for endotoxin contamination been added into the “Measurements of circulating endotoxin levels” paragraph (Please see Page 9).
5. LBP and sCD14 are two proteins that promote innate immunity to Gram-negative bacteria by transferring LPS to TLR4. Although LBP is synthesized by intestinal cells as well as the liver and adipose tissue, and sCD14 is secreted by the liver and monocytes, both are the potential biomarkers that could be correlated with LPS levels, and related to intestinal bacteria exposure. As the reviewer’s suggestion, LPS in blood is an important and feasible biomarker of intestinal barrier dysfunction. If other microbial translocation markers (e.g., LBP) are measured in the blood, it may provide stronger information for gut permeability. Agreeably, microbial translocation markers and sCD14 are considered to be measured for future related studies.

7. PLOS authors have the option to publish the peer review history of their article ([what does this mean?](https://journals.plos.org/plosone/s/editorial-and-peer-review-process#loc-peer-review-history)). If published, this will include your full peer review and any attached files.
If you choose “no”, your identity will remain anonymous but your review may still be made public.

**Do you want your identity to be public for this peer review?** For information about this choice, including consent withdrawal, please see our [Privacy Policy](https://www.plos.org/privacy-policy).

Reviewer #2: No

Reviewer #3: No
